# Synostosis of the Proximal Tibiofibular Joint

**DOI:** 10.1155/2010/794594

**Published:** 2010-06-08

**Authors:** Nikolaos K. Sferopoulos

**Affiliations:** 2nd Orthopaedic Department, Aristotle University of Thessaloniki, “G. Gennimatas” Hospital, 54635 Thessaloniki, Greece

## Abstract

The incidence of synostosis of the proximal tibiofibular joint (TFJ) was assessed among 1029 patients examined for osteoarthritis of the knee in a 4-year period. Radiographic evidence of a synostosis of the proximal TFJ was demonstrated in 3 knees (3 patients). The synostosis appeared incidental and was not the cause of symptoms in any of them. These patients were further examined with MRI and/or CT scans. In two cases, which were found to be primary (idiopathic), the synostosis was complete and bony. In a third case the lesion was secondary (acquired) to surgical reconstruction for a depressed fracture of the lateral tibial plateau. This iatrogenic complication followed open reduction, internal fixation, and grafting with synthetic bone. The bridging of the joint on the CT views was partial and compatible with ectopic calcification rather than ossification. The patients were treated conservatively and were followed for an average period of 3 years. No evidence that the synostosis accelerated the onset or progression of the degenerative changes to the ipsilateral knee could be verified.

## 1. Introduction

Synostosis of the proximal tibiofibular joint (TFJ) is extremely rare. It may occur in skeletally immature patients [1–3], adolescents [4], or adults [5–9]. It may be caused by the coalescence of “kissing” osteochondromata in patients with multiple hereditary exostoses [5, 6, 10], or may be associated with other generalized syndromes [7] and knee valgus [1–3]. Synostosis with no associated syndromes or deformities has been previously reported in only 3 patients. In two adults the synostosis was primary [8, 9], while in a 14-year-old girl the iatrogenic lesion was secondary to tibial nailing [4]. Our data referring to 3 patients with a unilateral synostosis of the proximal TFJ, identified among 1029 patients examined for degenerative knee arthritis, are presented and discussed.

## 2. Materials and Methods

 A total of 1029 patients, with an age range from 46 to 96 years (mean 71.34 years), were examined clinically and radiologically, in a 4-year period (from 2005 to 2008), for degenerative knee arthritis. There were 817 women with an age range from 46 to 94 years (mean 70.66 years) and 212 men with an age range from 46 to 96 years (mean 74.28 years). 

Synostosis of the proximal TFJ was diagnosed in 3 patients (3 knees). None of them was overweight and none of them complained of pain at rest or during the night. They all reported to have “normal” physical activities of daily living and no functional limitations. No symptoms could be detected around the proximal TFJ in any of our patients nor was there any demonstrable peroneal neuropathy. There was no observed difference in the patients with synostosis and the whole group of patients with degenerative arthritis in terms of pain, range of motion, activity level, and weather influences. According to the radiographic staging system of Kellgren and Lawrence [11], all our patients were classified as having grade I-II disease of the femorotibial joint. In two cases (1 and 2), the lesion was considered to be primary (idiopathic). The lesion was secondary (acquired) in case 3 following a reconstruction for a depressed lateral fracture of the tibial plateau, augmented with synthetic bone graft. In all cases, the right knee was affected. The two primary cases had an oblique-type proximal TFJ articulation. No abnormal features of the proximal TFJ on the controlateral side were radiologically observed. None of the patients had a history of a neoplasm, metabolic disorder, infection, rheumatoid arthritis, or steroid use. All patients received conservative treatment including viscosupplement intra-articular injections. No specific measures were undertaken for the synostosis of the proximal TFJ. Progression of the degenerative changes was evaluated after a follow-up period that ranged from 2 to 4 years (mean 3 years). Total knee arthroplasty was not undertaken by that time and will be delayed as long as the use of nonoperative methods is considered satisfactory. The 3 cases are reported below in details.

**Case 1**

A man, aged 55 years, complained of peripatellar aching and pain on the medial aspect of the joint, which increased on walking in both knees.

On palpation, patellar crepitation was noted with flexion and extension. Tenderness was present on the medial side of the joint over the medial collateral ligament and under the patella in both knees.

**Figure 1:**
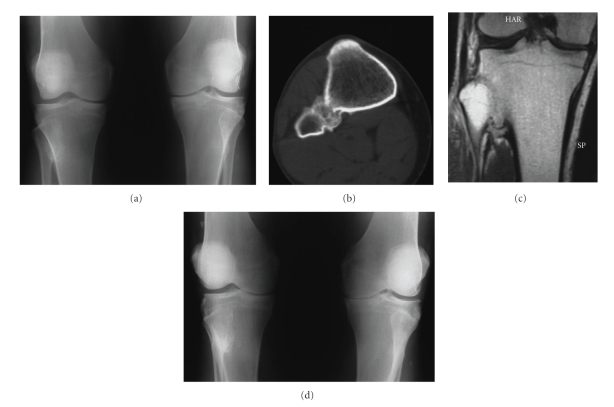
AP radiograph of both knees (a), CT (b), and MRI (c) examination, in a 55-year-old man, indicating synostosis of the proximal TFJ of the right knee initially, and 4 years later (d).

Anteroposterior and lateral standing radiographs of the long axis of both legs showed normal hip-knee-ankle alignment, no patellofemoral involvement, narrowing of the medial compartment space more pronounced on the left knee and synostosis of the proximal TFJ on the right knee (Figure 1(a)). The fibula had normal length and shape and the position of the proximal TFJ was normal on both sides. The synostosis was considered to be complete and bony by a CT scan (Figure 1(b)) and an MRI (Figure 1(c)). At 4-year follow-up the degenerative changes exhibit similar progression on both knees (Figure 1(d)). By that time, a severely reduced internal rotation of the right hip joint was diagnosed on clinical examination. Radiograph of the hips indicated narrowing of the joint space, subchondral sclerosis, cyst formation and osteophytes on the right side. No evidence of ankle arthritis was found.

**Case 2**

A 60-year-old woman was referred for pain on the medial aspect of both knees on walking. She had only slight pain going up and down stairs.

On palpation, tenderness was located on the medial side of the joints and over the insertion of the semimembranosus tendon in both knees.

**Figure 2:**
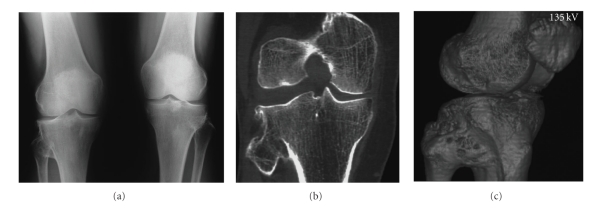
AP radiograph of both knees (a), CT (b) and 3D (c) examination, in a 60-year-old woman, showing synostosis of the proximal TFJ of the right knee.

**Figure 3:**
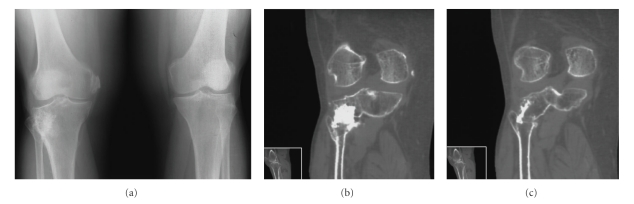
AP radiograph of both knees (a), and CT (b,c) examination in a 69-year-old woman, revealing ectopic calcification of the proximal TFJ of the right knee.

Anteroposterior and lateral standing radiographs of the long axis of both legs showed normal hip-knee-ankle alignment, no patellofemoral involvement, mild narrowing of the medial compartment space more pronounced on the right knee and synostosis of the right proximal TFJ (Figure 2(a)). The fibula had normal length and shape and the position of the proximal TFJ was normal on both sides. The synostosis was confirmed to be complete and bony by a CT (Figure 2(b)) and a 3D scan (Figure 3(c)).

Two years later her x-rays showed symmetrical progression of the degenerative changes. There was no evidence of osteoarthritic changes to the hips or ankles.

**Case 3**

A 69-year-old woman was presented with an 8-year history of a depressed fracture of the lateral tibial plateau of the right knee that was treated with open reduction, internal fixation, and was grafted with synthetic bone material. She complained of pain on walking in the right knee. She had also pain going up and down stairs.

On palpation severe tenderness was present on the medial side of the right knee, especially at the femoral insertion of the medial collateral ligament.

Anteroposterior and lateral standing radiographs of the long axis of both legs showed normal hip-knee-ankle alignment, no patellofemoral involvement, narrowing of the medial compartment space and a calcium deposit of the medial collateral ligament at its femoral insertion of the right knee (Pellegrini-Stieda disease). The fibula had normal length and shape and the position of the proximal TFJ was normal on both sides. A synostosis of the proximal TFJ on the right knee was also evident (Figure 3(a)). A CT scan confirmed partial synostosis that was characterized by the appearance of a large radiopaque mass in the area of the previously reduced and grafted fracture of the tibial plateau (Figures 3(b) and 3(c)).

At 3-year follow-up there was slight progression of the clinical findings and the radiographic degenerative changes. There was no evidence of hip or ankle osteoarthritis.

## 3. Discussion

Tibiofibular synostosis has occasionally been described in the literature. It may be evident in one of the three joints that the tibia and fibula share. 

Synostosis of the distal (inferior) TFJ is usually acquired-secondary to ankle fractures. It usually causes few symptoms and does not require any treatment in adults [12], while in children the fibular growth may be altered leading to valgus alignment of the ankle [13]. Synostosis of the middle TFJ (formed by the interosseous membrane) may be located at the level of the junction of the proximal and middle third of the tibia [14] or at the middle and distal third of the tibia [15]. Both cases that have been reported in the literature received surgical treatment. 

Synostosis of the proximal TFJ has been reported in both children and adults. Whenever the synostosis is present from birth or occurs before the closure of the proximal tibial growth plate, it is usually symptomatic and may be associated with other growth deformities [1, 2]. Predisposing factors may include the syndrome of multiple hereditary exostoses [5, 6, 10], generalized syndromes, like the 49, XXXXY karotype, while the role of knee valgus is unclear [7]. The absence of any growth abnormalities indicates that the synostosis occurred after physeal closure [9]. Only 3 such patients have previously been reported [4, 8, 9]. On the other hand, synostosis of the proximal TFJ in adults is rarely associated with complaints and may, therefore, be easily missed [4, 9].

 Two cases of symptomatic idiopathic synostosis of the proximal TFJ have been previously reported. One of them was a 24-year-old volley-ball player [9] and the other one was a 45-year-old man with intermittent peroneal neuropathy [8]. 

The only known reported case of asymptomatic secondary synostosis was a 14-year-old girl 5 years following tibial nailing [4]. 

All our patients had normal or near normal function and range of motion of the knees and also had no complaints due to the synostosis. The incidence of primary synostosis of the proximal TFJ in our series was 0.1% that is: 2 knees among 1029 patients (2058 knees). In our idiopathic cases (patients 1 and 2), the fibula had a normal length and shape, and the position of the proximal TFJ was normal. These findings indicated that the synostosis developed after the closure of the growth plates in adult life. The inclination of the TFJ surface in both these cases was of the oblique-type articulation, with an inclination of approximately 45 degrees. It is suggested that in the oblique-type articulation the load per surface area of the joint is increased resulting in higher pressure on cartilage [16]. This type of joint could also be more prone to degenerative changes [17]. However, it is questionable whether the appearance of synostosis may, in any way, be affected by the inclination of the proximal TFJ. 

The radiological appearance of the synostosis was complete and bony, and was similar in the idiopathic patients (cases 1 and 2), but was partial and appeared as a large radiopaque mass, compatible with ectopic calcification rather than ossification, in the acquired case (patient 3). The pathogenesis of this iatrogenic synostosis might be due to injury of the soft tissues and hemorrhage during surgery, or to the evacuation of a quantity of the synthetic bone graft. 

We found no evidence that the synostosis was in any way associated with the onset or progression of osteoarthritic changes to the ipsilateral knee. Furthermore, the radiological assessment of both knees indicated more severe degenerative changes on the ipsilateral knee on two cases (patients 2 and 3) and on the contralateral healthy side in another one (case 1). No osteoarthritic changes were found radiologically in the hips in two patients (cases 2 and 3), while there were severe changes of the ipsilateral hip in one patient (case 1). There was no evidence of ankle arthritis in our patients. 

In conclusion, the onset as well as the clinical and radiological progression of the degenerative changes of the knees, in all our 3 patients, was not, in any way, related to the unilateral proximal TFJ synostosis.
